# Multifocal Follicular Lymphoma Isolated to the Gastrointestinal Tract: A Rare Finding

**DOI:** 10.14309/crj.0000000000001215

**Published:** 2023-12-15

**Authors:** Alexander Garcia, Mahir Qureshi, Ishita Dhawan, Ashleigh Allen, Tulin Budak-Alpdogan, Samuel Giordano

**Affiliations:** 1 Department of Medicine, Cooper University Hospital, Camden, NJ; 2 Department of Gastroenterology, Cooper University Hospital, Camden, NJ; 3 Pathology, Cooper University Hospital, Camden, NJ; 4 Department of Hematology/Oncology, Cooper University Hospital, Camden, NJ

**Keywords:** follicular, lymphoma, small intestine, sigmoid colon

## Abstract

Follicular lymphoma (FL) is a common form of non-Hodgkin lymphoma. Although extranodal involvement of the gastrointestinal (GI) tract is common in lymphomas, primary GI-FL confined to the GI tract is relatively rare. The disease process is typically indolent in nature and usually incidentally found. Among this subset of patients, the duodenum and terminal ileum tend to be the most common site of origin. Here, we present a rare case of primary multifocal GI-FL that found incidentally during routine colonoscopy with subsequent esophagogastroduodenoscopy and video capsule endoscopy revealing duodenal, jejunal, and sigmoid colon involvement.

## INTRODUCTION

Follicular lymphoma (FL) is a common form of non-Hodgkin lymphoma. The gastrointestinal (GI) tract tends to be a common region of extranodal lymphomas. Although mucosal-associated lymphoid tissue lymphoma and high-grade B-cell lymphoma represent the most common forms of extranodal lymphomas, primary GI-FL is relatively rare with an estimated incidence rate of 1.0%–3.6%.^1,2^ Primary GI-FL typically involves most commonly the duodenum, specifically in the periampullary region, followed by the ileum and colon.^3^ In addition, with the increasing use of advanced endoscopic procedures, the literature has shown an increasing number of primary GI-FL detected in all regions of the small intestine.^4^ Of patients with primary GI FL, the majority tend to have multiple lesions throughout the GI tract.^5,6^ This article presents a middle-aged man with symptoms of heartburn who on endoscopic evaluation was found to have evidence of FL in the duodenum and sigmoid colon.

## CASE REPORT

**Figure 1.**
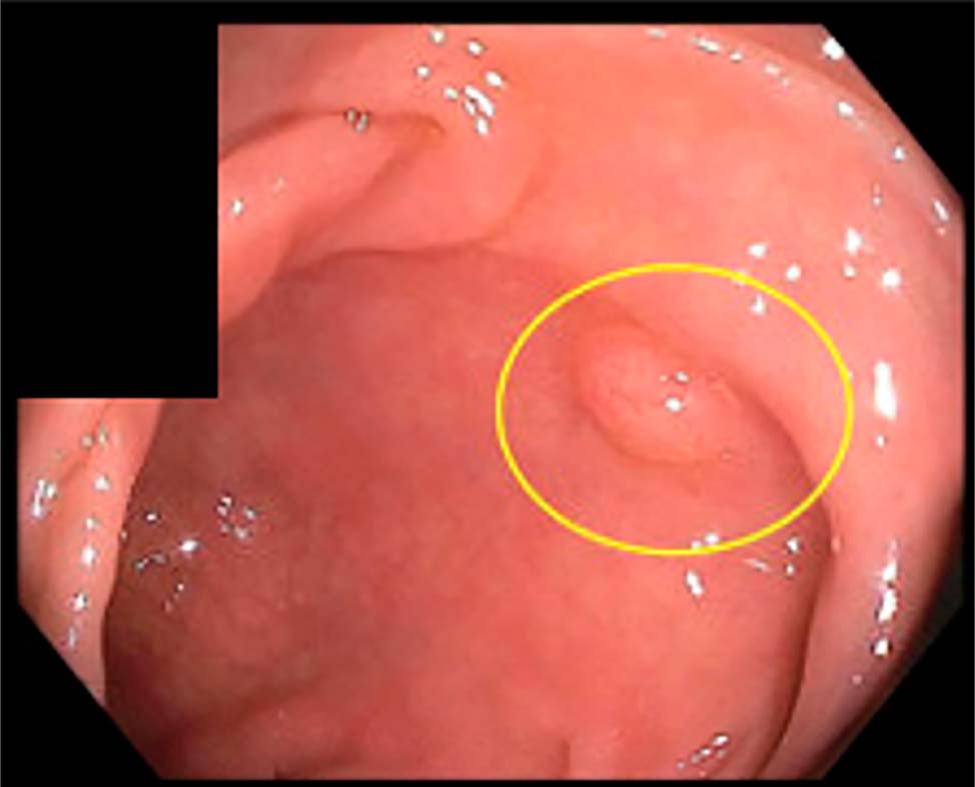
Polyp found to be positive for follicular lymphoma in the sigmoid colon.

A 59-year-old man with no significant medical history presented to the gastroenterology clinic for a screening colonoscopy. Before presentation, the patient had been asymptomatic without acute concerns. On colonoscopy, 4 colonic polyps were identified and retrieved for pathology including several 3–5 mm in the ascending, transverse, and sigmoid colon and one 7 mm polyp in the descending colon (Figure 1). The polyps found in the ascending, transverse, and descending colon were found to be tubular adenomas. However, the polyp in the sigmoid region was found to have prominent lymphoid aggregates, which was most consistent with FL.

**Figure 2.**
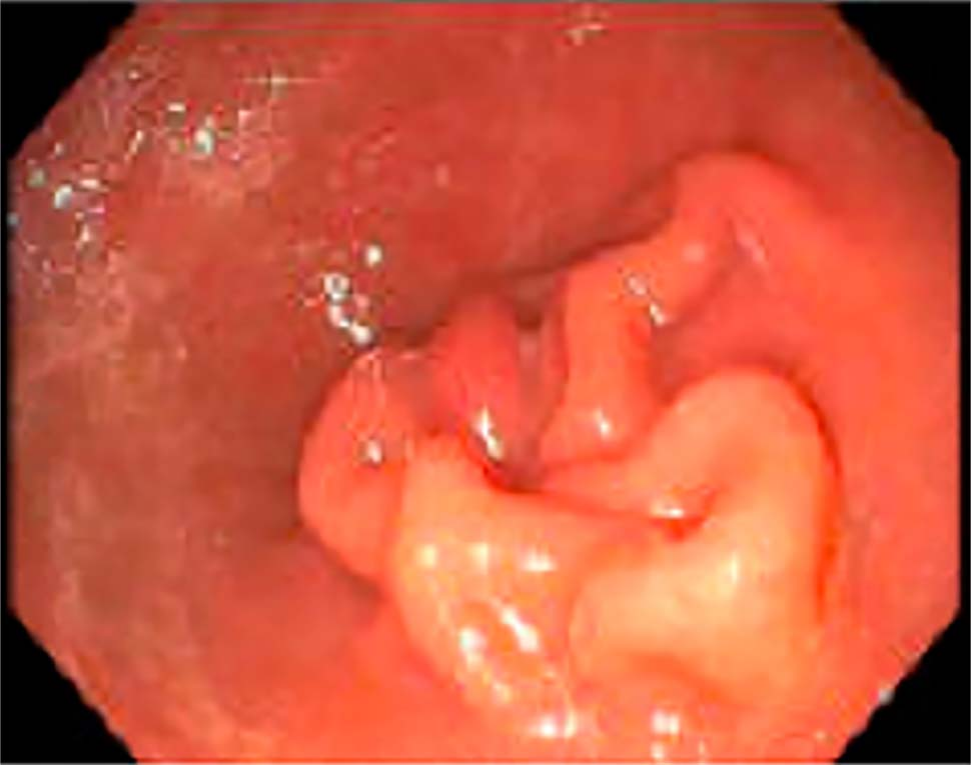
Polyp found to be positive for follicular lymphoma in the second part of the duodenum.

**Figure 3.**
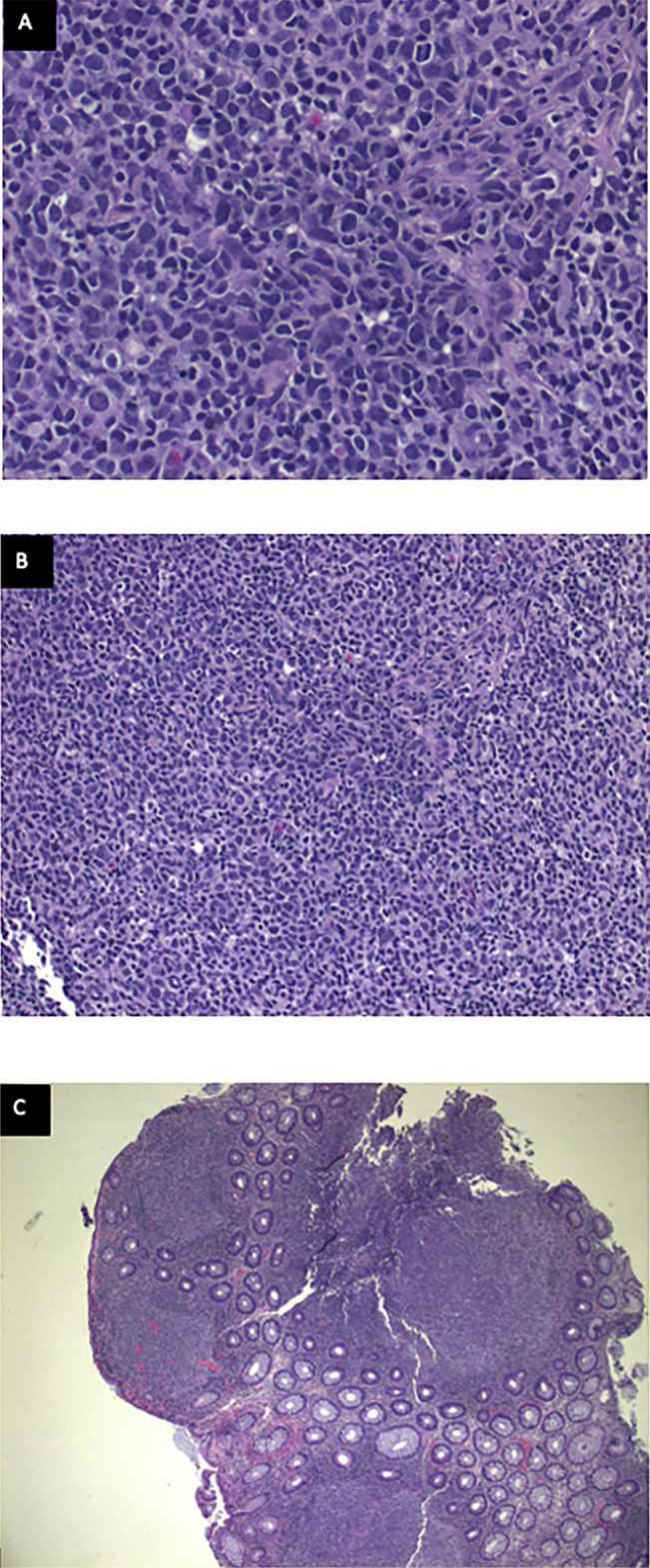
Multiple follicles with many angulated centrocytes and occasional, large centroblast in the colon in the highest magnification (A), high magnification (B), and low power magnification (C).

**Figure 4.**
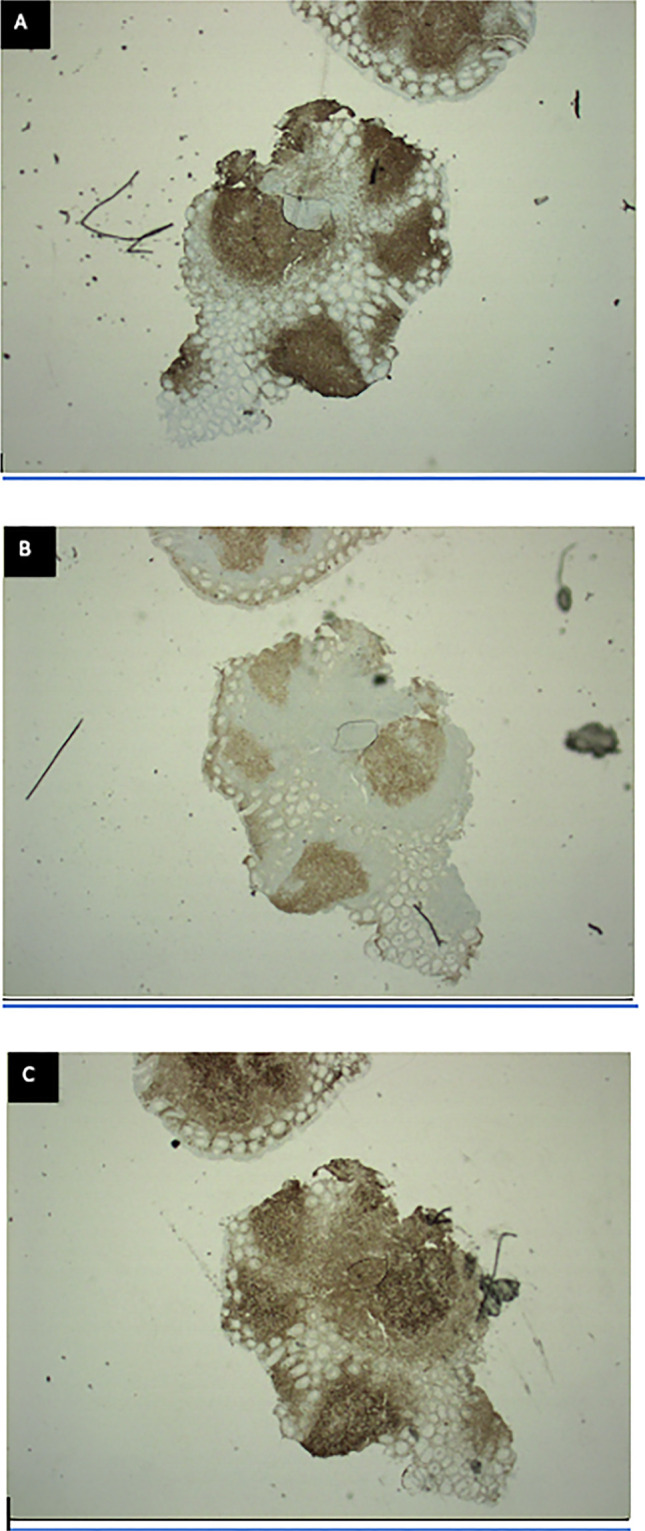
Follicles with evidence of CD-20 highlighting the B cells (A), CD-10 (B), and BCL2 positive (C) in the germinal centers.

**Figure 5.**
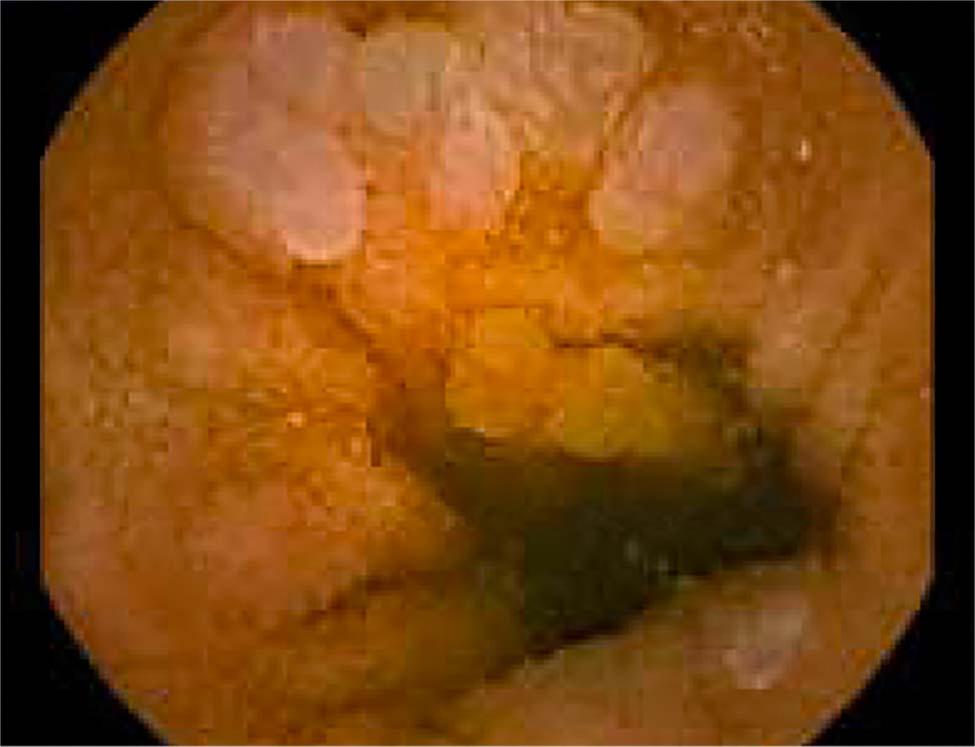
Video capsule endoscopy demonstrating nodularity in the mid to distal jejunum concerning for lymphoma.

On subsequent positron emission tomography (PET)/computed tomography (CT), no evidence of disease was evident outside the GI tract. Given the decreased sensitivity of PET/CT at identifying GI involvement, the patient underwent evaluation with esophagogastroduodenoscopy (EGD) to assess for additional sites of involvement. EGD demonstrated nodular duodenal mucosa that on microscopic analysis was found to be prominent lymphoid aggregates most consistent with FL as found in the sigmoid region on initial colonoscopy (Figure 2). Pathology demonstrated multiple follicles with angulated centrocytes and occasional, large centroblasts (Figure 3). Follicles were positive for CD20 for B cells, CD10 and BCL2 positive in the germinal centers, which is classic for FL (Figure 4). These lymphoid follicles, with atypical appearing germinal centers and irregular borders, were immunostain positive for CD20, PAX5, CD21, CD10, and BCL6 consistent with grade 2 FL at both sites. In addition, testing yielded a positive *t* (14; 18) and negative for *t* (11; 14) translocation. The work-up to rule out reactive lymphoid aggregates secondary to infection was negative for hepatitis C, hepatitis B, and HIV. Subsequent laboratory testing including a complete metabolic panel (including liver function tests) and complete blood count were normal. Video capsule endoscopy demonstrated nodularity in the mid to distal jejunum concerning for lymphoma (Figure 5). Follow-up with oncology is scheduled.

## DISCUSSION

Duodenal-type FL is a newly recognized entity in the 2016 World Health Organization classification update.^7^ Primary GI-FL is typically found incidentally during routine endoscopic examination. Patients tend to be asymptomatic on presentation, but few patients have reported abdominal pain, nausea, vomiting, and gastrointestinal bleeding.^5,8,9^ The literature reports a 2:1 female predominance and an average age of diagnosis 66 years or older.^10^ Generally, this disease manifests itself endoscopically as small polypoid lesions coated in normal mucosa but can rarely present itself as ulcers with subsequent stenosis of the intestinal lumen. The pathogenesis remains unclear; however, there have been reported cases describing evidence of regression in patients with duodenal FL who were positive and successfully treated for *Helicobacter pylori*.^11,12^

Interestingly, our patient was found to have GI-FL with involvement of both the duodenum and sigmoid colon. Generally, lymphomas of the GI tract commonly involve the stomach and small intestine (duodenum being the leading site of involvement), with colorectal lymphomas representing a smaller proportion. In fact, sigmoid FL comprises approximately 0.2%–1% of colonic malignancies.^13^ To our knowledge, few cases exist describing GI-FL in the duodenum, jejunum, and sigmoid colon simultaneously making our case unique. Staging of the malignancy is also difficult because there is no evidence of any definitive lymph node involvement currently.

As in our patient, this disease process exhibits an immunophenotype similar to other FLs and carries the classic *t* (14; 18) (q32; q21) translocation. Patients who have confirmed pathological evidence of primary GI-FL on endoscopic examination are recommended to undergo a thorough work-up consisted of further endoscopic examination (EGD/video capsule endoscopy), a chest and abdominal CT scan, a PET scan, and a bone marrow biopsy.^14^ Although considered the golden standard for diagnosis of extranodal disease, PET/CT can often lead to false negatives. Our patient was found to have no evidence of extranodal involvement on the PET scan, including the GI tract, despite having biopsy-proven colonic disease. One study showed the sensitivity and false negative tracer uptake of PET/CT to be 46.3% and 53.7%, respectively, for primary GI-FL,^15^ highlighting the importance of a thorough work-up when investigating disease burden.

Most importantly, the prognosis differs for primary colorectal FL and duodenal FL. The former has a low median survival rate at a range from 24 to 36 months with high rates of recurrence despite undergoing systemic chemotherapy.^16,17^ However, 5-year progression-free survival for primary duodenal FL has been as high as 98%.^9^

Treatment depends on the progression and extent of spread of the disease. For unifocal primary GI-FL without poor prognostic factors, radiotherapy can be used. If radiotherapy is contraindicated, monotherapy with rituximab can be used. If a poor prognosis is present and/or disease is multifocal, systemic therapy with or without rituximab can be used. Long-term outcome of GI-FL is unknown because of the rarity of this disease. Treatment may not be needed in the absence of symptoms or disease progression.^18^ As our patient remains asymptomatic, treatment has not been initiated.

Multifocal primary GI-FL is particularly a rare malignancy that is not completely understood but tends to follow an indolent pattern and typically incidentally found. When pathological evidence is present, a thorough work-up is warranted to determine the severity and extent of this disease. Treatment varies depending on the focality of the lesion(s) and the presence of symptoms.

## DISCLOSURES

Author contributions: A. Garcia, M. Qureshi, I. Dhawan: created and reviewed the manuscript. A. Allen: read and analyzed the pathology. T. Budak-Alpdogan, S. Giordano: reviewed the final manuscript. S. Giordano is the article guarantor.

Financial disclosure: None to report.

Informed consent was obtained for this case report.
